# Stratifying high-risk prediabetes clusters using blood-based epigenetic markers

**DOI:** 10.1186/s40364-025-00887-8

**Published:** 2026-01-17

**Authors:** Amandeep Singh, Reiner Jumpertz-von Schwartzenberg, Robert Wagner, Leontine Sandforth, Arvid Sandforth, Markus Jähnert, Marlene Ganslmeier, Stefan Kabisch, Nikolaos Perakakis, Hubert Preißl, Andreas Fritsche, Norbert Stefan, Dirk Walter, Meriem Ouni, Andreas L. Birkenfeld, Annette Schürmann

**Affiliations:** 1 Department of Experimental Diabetology, German Institute of Human Nutrition Potsdam-Rehbruecke (DIfE), Arthur-Scheunert-Allee 114-116, 14558 Nuthetal, Germany; 2 Research group epigenetic of obesity and diabetes, German Institute of Human Nutrition Potsdam-Rehbruecke (DIfE), Nuthetal, Germany; 3 German Center for Diabetes Research (DZD), München-Neuherberg, Germany; 4 Institute for Diabetes Research and Metabolic Diseases (IDM) of the Helmholtz Center Munich, University of Tübingen, Tübingen, Germany; 5 Internal Medicine IV, Department of Endocrinology, Diabetology and Nephrology, Eberhard Karls University Tübingen, Tübingen, Germany; 6 Department of Endocrinology and Diabetology, Medical Faculty and University Hospital Düsseldorf, Heinrich Heine University Düsseldorf, Düsseldorf, Germany; 7 Institute for Clinical Diabetology, German Diabetes Center, Leibniz Center for Diabetes Research at Heinrich Heine University Düsseldorf, Düsseldorf, Germany; 8 Clinic of Endocrinology and Metabolism, Charité Universitätsmedizin Berlin, 10117 Berlin, Germany; 9 Helmholtz Center Munich, Faculty of Medicine, Paul Langerhans Institute Dresden, University Hospital, Technische Universität Dresden, Dresden, Germany; 10 Department of Internal Medicine III, University Hospital Carl Gustav Carus, Technische Universität Dresden, Dresden, Germany; 11 Max Planck Institute of Molecular Plant Physiology, Am Mühlenberg 1, 14476 Potsdam, Golm, Germany; 12 Institute of Nutritional Science, University of Potsdam, Potsdam, Germany

**Keywords:** Prediabetes, Risk stratification, Epigenetic (bio)-markers, Prognostic tools, Machine learning

## Abstract

**Background:**

Previously, we identified six prediabetes clusters, three at moderate and three at high-risk for type 2 diabetes and/or complications. While this novel classification could enable earlier and improved disease prevention, it relies on intensive clinical phenotyping. Here, we developed a machine learning workflow to identify blood-based epigenetic markers to distinguish between prediabetes clusters.

**Methods:**

DNA methylation was profiled in blood cells of different cohorts including individuals that belong to clusters 2 (low-risk), 3, 5, and 6 (each high-risk) and data was subjected to a machine learning workflow.

**Results:**

In a discovery cohort (*n* = 187), we identified 1,557 CpG sites as predictors for clusters 2, 3, 5, and 6. These CpGs were sufficient to distinguish between individuals belonging to the high-risk clusters 3, 5 and 6 in an independent replication cohort (*n* = 146) with an accuracy of 92%. Between 300 and 339 CpG sites were specific for each cluster and the corresponding genes linked to TGF-β receptor and calcium signaling (cluster 3), MAPK cascade and ECM organization (cluster 5), and Wnt/SMAD signaling (cluster 6), mirroring the metabolic deterioration observed in each cluster.

**Conclusions:**

Without the need for complex clinical measurements, the identified blood-based epigenetic signatures may improve the detection of individuals at high-risk of developing diabetes and complications and point to the potential molecular mechanism responsible for the heterogeneity in prediabetes. These markers highlight the potential of the blood epigenome as an effective proxy for predicting future complications and make extensive clinical assessments obsolete, enabling the identification of clusters in larger populations.

**Supplementary Information:**

The online version contains supplementary material available at 10.1186/s40364-025-00887-8.

## Background

Type 2 diabetes (T2D) is one of the top ten causes of death worldwide, making its prevention an urgent clinical need. The high mortality risk associated with T2D is mainly due to the development of severe complications such as cardiovascular diseases, nephropathy, and cancer [1–3].

Identifying people with an elevated diabetes risk has practical implications, as there are interventions that can prevent or delay T2D onset, and earlier disease diagnosis may improve clinical and economic burden [4, 5]. The development of T2D is a continuous process from normal glucose regulation to prediabetes and T2D. Several researchers considered prediabetes as a target phase for biomarker screening, to improve early detection, facilitate more effective interventions, and ultimately reduce the global burden of T2D and its complications [4, 6, 7]. We showed recently that in adults with impaired fasting glucose and/or glucose tolerance, a one-year lifestyle intervention with ≥ 5% weight loss led to prediabetes remission (i.e. normalization of fasting and 2 h glucose during an oral glucose tolerance test and of HbA1c) in 43% of the participants. This was associated with a significant reduction in the risk to develop future T2D [8]. Thus, remission of prediabetes should be an important goal to prevent development of severe metabolic diseases [6, 9]. However, individuals with prediabetes are a heterogeneous group and prediabetes is driven by one or more factors such as elevated fat mass, obesity, insulin resistance, impaired insulin secretion and low grade of inflammation [10].

Recently, data-driven clustering strategies have enabled a better understanding of the heterogeneous pathophysiology of prediabetes [11]. Six distinct clusters, the Tübingen Diabetes Risk Clusters, were identified, each with varying risks for developing T2D and associated complications. Three of these (clusters 1, 2, and 4) are characterized by a low-risk of diabetes progression, whereas clusters 3 and 5 exhibited the highest T2D incidence; the T2D risk of cluster 6 was only slightly higher than of that of low-risk cluster 1, however, this group had a high-risk for chronic kidney disease and mortality and was characterized by elevated visceral fat. Individuals in cluster 3 had lowest insulin secretion, cluster 5 had increased intrahepatic lipid content and high insulin resistance. Both, clusters 3 and 5, also demonstrated higher risks for chronic kidney disease relative to cluster 1. Overall, cluster 5 exhibited the highest risk of T2D, renal and vascular disease and all-cause mortality (Suppl Fig. 1a). To easily connect these clusters to their metabolic differences, we included additional abbreviations to the original nomenclature and used them interchangeably in this study. For instance, low risk (LR or cluster 2), high risk low beta cell function (HR-LowBeta or cluster 3), high risk high insulin resistance (HR-InsRes or cluster 5), high risk high insulin secretion (HR-InsSecr or cluster 6). Although this sub-phenotyping approach offers potential improvements in screening, prevention, and treatment strategies, it remains complex and difficult to apply in large clinical settings. Thus, there is an urgent need for the development of simple clinical tests or the identification of biomarkers detectable in easily accessible material.

Our group and others have found epigenetic signatures associated with obesity and T2D in different tissues, suggesting that epigenetic mechanisms contribute to the pathogenesis of T2D [12–14]. We also showed that blood-based epigenetic markers (e.g. DNA methylation) are a valuable proxy for risk assessment in T2D [15, 16].

However, it remains unknown whether DNA methylation patterns differ between the prediabetes clusters introduced recently by us [11]. Hence, the present study investigated the DNA methylome in peripheral whole blood cells in order to identify epigenetic markers distinguishing individuals from high- (clusters 3, 5, and 6) and a low-risk cluster (cluster 2).

## Methods

### Study population description

The individuals included in the present study are a subgroup of participants from the TUEF/TULIP cohort, the Prediabetes Lifestyle Intervention Study (PLIS) (ClinicalTrials.gov identifier: NCT01947595) and the IFIS study (ClinicalTrials.gov identifier: NCT04607096) all of which included adults with prediabetes. Individuals were selected based on completeness of data and the closest location to the respective cluster medoids.

The current study included two study cohorts: a discovery (*n* = 187, Tübingen/Dresden) and a replication cohort (*n* = 146 Potsdam/Tübingen). The characteristics of the participants in the discovery and replication cohorts are detailed in Suppl Fig. 1a, Table 1 and S1. Clinical and metabolic measurements are described in Supplementary Material 2. Blood samples for DNA methylation measurements were drawn prior to the oral glucose tolerance test (OGTT) at baseline study visits. The cluster membership of all individuals in the discovery cohort was confirmed with both the TUEF/TULIP and the simplified Whitehall criteria as has been previously described [11].

**Table 1:** Participants’ characteristics. Data are presented as median [IQR]

	Discovery Cohort					Replication Cohort			
	Cluster 2	Cluster 3	Cluster 5	Cluster 6	P-value	Cluster 3	Cluster 5	Cluster 6	P-value
	(*N* = 50)	(*N* = 42)	(*N* = 45)	(*N* = 50)		(*N* = 49)	(*N* = 51)	(*N* = 46)	
Sex (female/male)	32/18	22/20	28/17	23/27	0.235	40/9	40/11	38/8	0.859
Age [years]	59.5 [50.1–65.0]	64.36 [55.3–67.0]	58.4 [45.0–64.0]	52.50 [34.0-60.7]	< 0.001	59.4 [52.6–63.3]	58.6 [52.5–63.7]	59.4 [52.2–65.0]	0.928
BMI [m/kg^2]	24.2 [21.8–26.1]	28.2 [25.6–31.2]	34.3 [29.6–42.4]	35.2 [31.6–38.1]	< 0.001	30.0 [26.5–31.9]	34.5 [32.4–38.5]	35.0 [31.0-40.9]	< 0.001
IHL [%]	1.48 [0.86–2.76]	5.92 [1.60-12.54]	13.98 [7.53–18.15]	7.53 [2.92–11.80]	< 0.001	4.66 [2.27–8.27]	20.38 [15.03–23.08]	8.22 [6.00-11.44]	< 0.001
VAT [l]	1.99 [1.40-3.00]	5.40 [3.54–7.07]	5.78 [4.92–7.85]	4.46 [3.95–7.55]	< 0.001	3.79 [2.79–4.84]	5.24 [4.19–6.57]	5.18 [4.29–6.80]	< 0.001
Matsuda	5.68 [4.03–6.76]	3.05 [2.48–4.19]	1.54 [1.21–2.10]	1.84 [1.45–2.40]	< 0.001	3.00 [2.19–3.59]	1.31 [0.96–1.73]	1.82 [1.28–2.36]	< 0.001
Disposition Index	3.17 [2.15–5.11]	1.88 [1.31–3.01]	1.59 [1.08–2.50]	3.20 [1.47–3.98]	< 0.001	1.63 [1.18–2.43]	1.59 [1.05–1.94]	3.04 [2.10–4.01]	< 0.001
HbA1c [%]	5.7 [5.5–5.9]	5.9 [5.7-6.0]	5.9 [5.7–6.1]	5.9 [5.5-6.0]	0.019	5.8 [5.5–5.8]	5.9 [5.6–6.1]	5.8 [5.6-6.0]	0.025
Triglycerides [mg/dl]	76 [56–95]	132 [103–174]	161 [120–201]	124 [102–157]	< 0.001	110 [86–131]	166 [121–222]	113 [103–153]	< 0.001
Cholesterol [mg/dl]	206 [181–225]	206 [179–241]	208 [182–238]	195 [171–206]	0.196	210 [188–226]	209 [185–235]	207 [180–227]	0.653
HDL [mg/dl]	70 [56–76]	53 [47–61]	45 [40–50]	45 [38–53]	< 0.001	55 [47–63]	47 [40.50–53.50]	51 [41–58]	0.008
LDL [mg/dl]	110 [95–134]	134 [100–164]	128 [104–169]	123 [101–141]	0.030	125 [104–140]	134 [113–156]	124 [106–148]	0.244
GOT [U/l]	23 [19–29]	28 [22–33]	25 [21–29]	26 [21–29]	0.273	24 [20–27]	26 [22–29]	25 [22–28]	0.083
GPT [U/l]	21 [18–28]	30 [23–38]	33 [24–46]	32 [24–39]	< 0.001	21 [17–30]	31 [25–42]	28 [22–37]	< 0.001
Creatinine [mg/dl]	0.80 [0.62–0.80]	0.80 [0.68-1.00]	0.70 [0.60–0.82]	0.84 [0.80–0.98]	0.002	0.80 [0.70–0.80]	0.80 [0.70–0.82]	0.70 [0.60–0.80]	0.181
CRP [mg/dl]	0.05 [0.02–0.14]	0.17 [0.10–0.62]	0.51 [0.14–1.23]	0.54 [0.15–1.17]	< 0.001	0.17 [0.08–0.46]	0.29 [0.16–0.58]	0.29 [0.13–0.57]	0.012

### Genome-wide DNA methylation profiling

Genomic DNA was isolated from blood cells via precipitation with sodium acetate. DNA methylation levels, after bisulfite treatment, were determined by the Infinium MethylationEPIC BeadChip (EPICv1.0, 850 K & EPICv2.0, 930 K). Bisulfite treatment and array hybridization were assessed by Eurofins Genomics Europe shared services GmbH and LIFE & BRAIN GENOMICS GmbH. The data were processed using R (v.4) packages “meffil” (v.1.3.1 & v.1.3.6) and “ChAMP” (v. 2.24.0 & v.2.29.1).

The raw data from each cohort was processed independently using the default parameters of the champ.load() function, which performs standard quality control. Background correction and three-state β-mixture quantile normalization were followed by batch effect correction (including technical confounders) based on the singular value decomposition (SVD) output (p-values) as well as correction for cell composition. Data were normalized for age and sex using ComBat. DNA methylation data is represented as β (range 0–1); the β value of zero signifies no DNA methylation at the corresponding CpG position and one signifies 100% DNA methylation. Next, a variance filter was applied to remove 235,455 CpG sites displaying consistently low (< 5%) or high (> 95%) methylation levels within each cohort, as these sites typically exhibit very limited variability in blood cells. In total, differential methylation analysis was performed for a total of 442,248 CpG sites (CpGs) using the ChAMP package.

### Machine learning-based feature selection

To identify a robust set of predictive methylation sites for the prediabetes clusters, a feature selection procedure based on Elastic-net (E-net) regularization was implemented. In the discovery cohort, 70% (*n* = 132) of randomly selected individuals were assigned to the training set, and the remaining 30% (*n* = 55) to the test set. The splitting procedure (70% training, 30% testing) was repeated 1,000 times to stabilize the feature selection and avoid potential confounders. For each split, E-net logistic regression was applied to the training data using the R glmnet (version 4.1-8) package [17]. Using E-net, the optimal regularization parameter (λ) was identified through 5-fold cross-validation, and alpha (α), the L1/L2 norm mixing factor, was identified using a grid search within the training data [18]. The predictive performance of each trained model was then evaluated on the corresponding held-out discovery-test set (also called: prediction of clusters labels). All models achieving a prediction accuracy > 80% were considered successful, and their corresponding features were retained. Finally, a consensus feature set was derived by selecting only those CpGs that were identified in at least 10% of the models, thereby prioritizing features demonstrating consistent predictive power across multiple data splits and different model training runs (detailed in Suppl Fig. 1b).

### Partitioning around medoids (PAM)

As the original phenotypic clusters were generated with an unsupervised method [11], we applied a similar approach to group individuals based only on methylation patterns using R package: cluster (v2.1.8) [19]. Following feature selection, the CpGs identified by multi-fold E-net were subjected to unsupervised clustering (partitioning around medoid; PAM) in the discovery and in an independent replication cohort to assess the performance efficiency of the CpG predictors.

### Pathways enrichment analysis

All CpG sites identified as predictors for prediabetes clusters differentially methylated CpG were annotated to the corresponding gene based on their location and the region within the genome. All CpG sites located within 2 kb upstream and/or downstream of the transcription starting site (TSS) are annotated to the promoters of the nearest genes; however, ones located in intergenic regions were excluded from this step. CpG sites within the range of the first to the last exon are considered as gene body. Gene ontology enrichment analysis was performed using DAVID version v2024q2. The cut-off parameters for DAVID include p-value < 0.05 and a minimum number of genes in each category as five.

### Epigenome-wide association studies (EWAS)

In order to compare the CpG sites selected by E-net to the previous T2D incidence findings, we used one of the most comprehensive data bases available: MRC-IEU catalogue. Data from the MRC-IEU catalogue of epigenome-wide association studies [20–29] was downloaded and filtered for studies conducted in blood cells. Traits related to obesity, T2D, C-reactive protein, lipids, heart and kidney diseases have been selected and re-categorised in broader terms (detailed description in supplementary data/tables, sheet “Filter_Criteria”).

### Statistical analyses

The clinical characteristics of the individuals were shown as median and interquartile range (IQR) and mean ± SD (Table 1 and S1). One-way ANOVA was used to assess differences among the groups (> 2). The two-sample Wilcoxon rank test or Unpaired t-test was used based on the data normality tested by Shapiro Wilk normality test and variance with F test. For the categorical data, the Chi-square test was used, and for the analyses, *p* < 0.05 was considered significant. For the differential methylation analysis, default ChAMP parameters were used and adjusted P-values (AdjP < 0.05; Benjamini Hochberg test) was considered statistically significant. The Spearman test was performed to check the correlation between the subset of CpGs (beta values) and the clinical and anthropometric measurements. Correlations with raw p-value below 0.05 were considered statistically significant.

## Results

### Study design and population characteristics

**Fig. 1:**
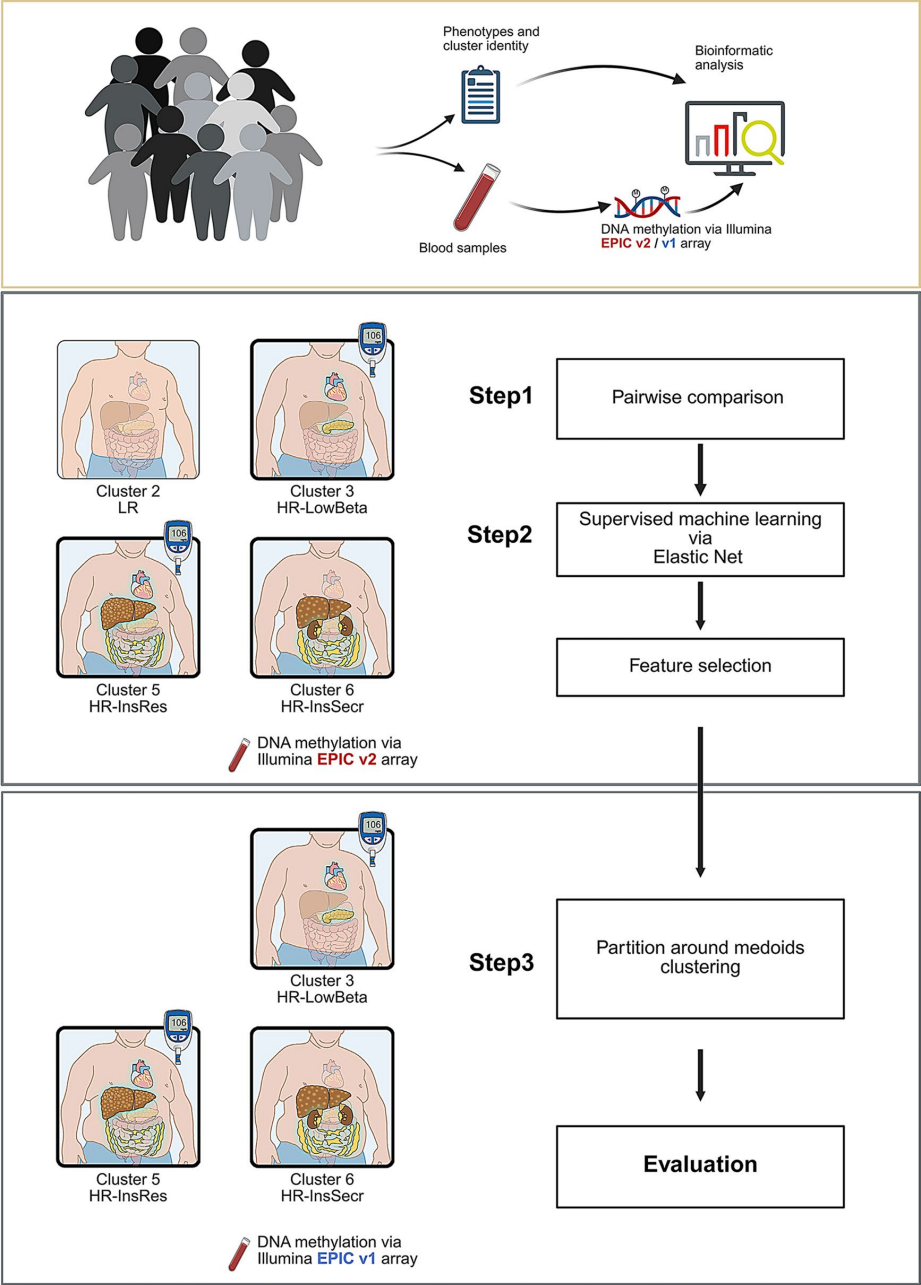
Schematic representation summarizing different steps of the current study and the cohorts. Two independent groups of participants are included; a discovery and a replication cohort. The cluster identity of the different participants was evaluated by criteria described in our previous study [11]. First, a pairwise comparison was conducted to define epigenetic signatures associated with: Cluster 2 (low risk: LR), Cluster 3 (high risk/low beta cell function: HR-LowBeta), Cluster 5 (high risk/high insulin resistance: HR-InsRes), Cluster 6 (high risk/high insulin secretion: HR-InsSecr). Next, a machine learning pipeline was applied to all differentially methylated CpGs to identify CpGs as predictors of prediabetes clusters. Results of the machine learning analyses identified in the discovery cohort were validated in an independent replication cohort with PAM clustering (more details are available in Suppl Fig. 1b)

The discovery cohort included 50 individuals belonging to cluster 2 (LR), 42 to cluster 3 (HR-LowBeta), 45 to cluster 5 (HR-InsRes), and 50 to cluster 6 (HR-InsSecr), as defined by the Tübingen Diabetes Risk parameters. The replication cohort was exclusively composed of individuals from the high-risk clusters HR-LowBeta, HR-InsRes, and HR-InsSecr (Fig. 1). As detailed in Table 1 and S1, the anthropometric and metabolic parameters of participants from the discovery (*n* = 187) and the replication cohort (*n* = 146) show that individuals in cluster 5 (HR-InsRes) had the highest BMI, VAT, IHL and also highest glucose and insulin levels. On the contrary, people of cluster 2 (LR) were leaner, had the lowest VAT mass, IHL, and glucose levels. In the high-risk clusters, people of cluster (HR-LowBeta) were slightly more insulin-sensitive and leaner than those of the other high-risk clusters, and cluster 6 (HR- InsSecr) showed the highest Disposition Index as a measure for beta cell function. These data confirm the characteristics specific to the respective prediabetes clusters as previously reported [11].

### DNA methylation signatures predicting prediabetes sub-phenotypes

Figure 1 illustrates the study design, the different steps, and cohorts (discovery, replication) used to screen for circulating epigenetic markers. In step 1, we used pairwise comparisons (comparing cluster 2 with 3, 5, 6; cluster 3 with 2, 5, 6; cluster 5 with 2, 3, 6; and cluster 6 with 2, 3, 5) in the discovery cohort to detect differentially methylated CpGs between these clusters (Fig. 1, Suppl Fig. 1b), which were combined as an input for the machine learning workflow for the identification of CpG predictors of the prediabetes clusters (step 2, Fig. 1). We identified 120,917 sites with differences in DNA methylation between any of the four clusters (also referred to as DMPs, differentially methylated positions; *n* = 187, AdjP < 0.05). Using all DMPs – independent of their direction of change in the methylation level – resulted in an insufficient separation of the clusters, indicating the presence of noise and uninformative signals (Suppl Fig. 2). The reason for this may be the discrepancy between the relatively low sample size, the number of the tested groups and the high dimensionality of the methylation data.

In order to reduce the noise and high dimensionality of the methylation data, DMPs were deployed as an input for multi-fold E-net to select features (differentially methylated CpGs) with high potential to distinguish between the different groups. This effectively reduced the dimensionality and identified the most informative DMPs for cluster discrimination. We generated 1,000 E-net models (1,000 different combinations of training and testing data sets) and all these models had an accuracy higher than 80%. In total, these 1,000 models included 22,404 CpG predictors (detailed in Suppl Fig. 1b), of which 1,557 CpGs appeared to be the most stable, as they were found in at least 10% of the successful models. All in all, the 1,000 E-net models showed consistently high performance, with both balanced and unbalanced accuracies exceeding 95% for all clusters (Suppl Fig. 3). Additionally, the high sensitivity, specificity (> 0.95) and F1 scores (> 0.90) further confirm the stability and generalizability of the selected features.

**Fig. 2:**
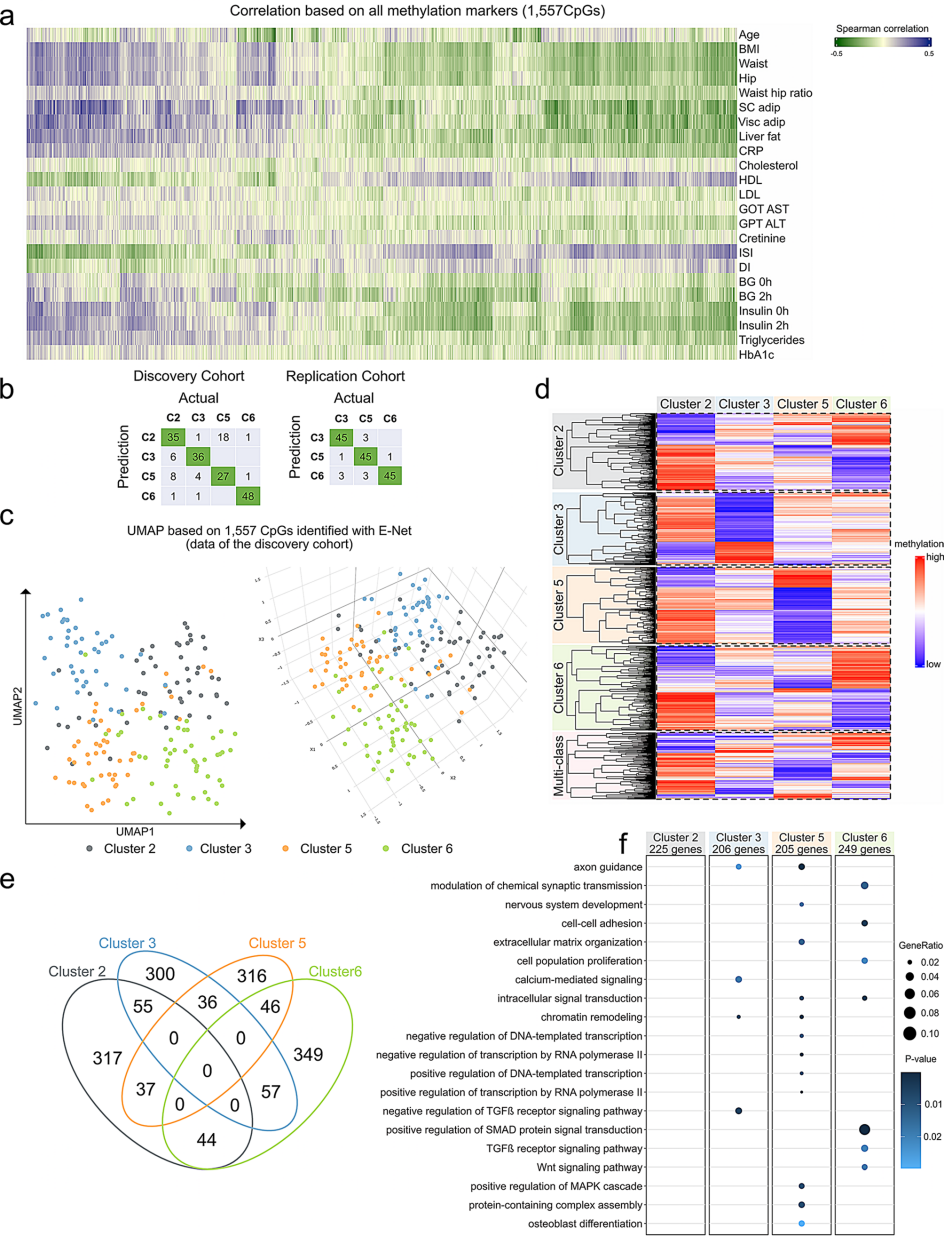
DNA methylation signatures in prediabetes clusters. (**a**) Spearman correlation between DNA methylation levels detected in the 1,557 CpG sites and metabolic parameters (shown in Table 1) are assessed in the discovery cohort and filtered for significance (raw *p* < 0.05). Correlation coefficients are depicted in a heatmap (blue: positive correlation; green: negative correlation coefficient). (**b**) Results of the partition around medoids clustering approach comparing predicted (methylation driven) and actual (phenotype driven) cluster identity. The agreement between methylation and phenotype-driven clustering is shown in the green rectangles. Grey rectangles refer to the disagreement between the two clustering. (**c**) UMAP plots separate the four clusters with high accuracy (left: 2D UMAP plot, right: 3D UMAP plot). (**d**) The heatmap depicting scaled DNA methylation levels of 1,557 CpG sites identified via machine learning algorithm in the discovery cohort (red: high level of DNA methylation coefficient; low level of DNA methylation) in each column is shown mean DNA methylation per group and each line refer to one CpG sites. (**e**) The different overlaps between CpGs predicting each cluster are summarized in the Venn diagram. (**f**) Dot plot depicting the results of GO analysis performed with DAVID tools, p-value < 0.05. Detailed GO terms are found in Table S3

To assess whether these CpG predictors reflect the clinical traits originally used to generate the clusters, spearman correlation was applied to the 1,557 CpGs with the participants’ anthropometric and metabolic measurements (Table 1). Out of these 1,557, 1,512 CpGs correlated with at least one of the examined traits as shown in the heatmap in Fig. 2a. The highest number of correlations were detected for ISI Matsuda index (882 CpGs), insulin (881 CpGs), and blood glucose concentration during OGTT (642 CpGs). Not only glycemic parameters but also blood lipids were correlated with methylation markers; 630 CpGs with triglycerides and 627 CpGs with HDL cholesterol. MR-tomography-based measurements of body fat exhibited significant correlations with 602 CpGs for the subcutaneous adipose tissue, 498 for the visceral adipose tissue, and 658 CpGs with liver fat. Additionally, a large number of CpGs were correlated with BMI (949 CpGs), however, only 305 CpGs were linked to age (Fig. 2a, Table S2).

Partitioning, based only on DNA methylation levels of the 1,557 CpGs, successfully reproduced the cluster identities of participants from the discovery cohort (accuracy = 78.07%), in the replication cohort (accuracy = 92.4%) (Step 3 in Figs. 1b and 2b-c). To further confirm the specificity of the identified methylation sites, we assessed the performance of a randomly selected subset of 1,500 CpGs after PAM clustering and repeated the procedure fifteen times. Accuracy based on those random CpGs were all lower than 60% as shown in Suppl Fig. 4. Thus, this machine learning algorithm is sufficient to assign the groups defined by DNA methylation into the correct prediabetes clusters as classified by clinical parameters. Here it has to be highlighted that the DNA methylation patterns of the replication cohort was generated by EPIC-v1 array, indicating that the introduced procedure can even be applied to older data generated by the earlier EPIC version (step 3 in Fig. 1).

The methylation patterns of the 1,557 CpGs predictors, which map to 1,021 genes, were visualized using the dimensionality reduction method, the uniform manifold approximation and projection (UMAP) as well as in a heatmap. These analyses revealed a unique methylation signature for each cluster. As shown in the UMAP plot (Fig. 2c) and in the heatmap (Fig. 2d), we could clearly distinguish between clusters 2 (LR), 3(HR-LowBeta), 5 (HR-InsRes) and 6 (HR-InsSecr) based only on the methylation levels of the identified 1,557 CpG sites. Of note, the DNA methylation patterns of these CpG predictors were different and distinguishable between the four prediabetes clusters (Fig. 2d). Among these, different subsets of CpGs showed unique methylation patterns in each cluster (marked by frames in the heatmap Fig. 2d). As revealed in the Venn diagram, 317 CpGs were specific for cluster 2 (LR), 300 for cluster 3 (HR-LowBeta), 316 for cluster 5 (HR-InsRes), and 349 for cluster 6 (HR-InsSecr) (Fig. 2e).

Overall, close to 75% of these CpG predictors were specifically hypermethylated in cluster 2, 70% hypomethylated in cluster 5, whereas cluster 6 showed a higher proportion (~ 60%) of hypermethylated CpGs especially when compared to cluster 5 and cluster 3 (Fig. 2d). To assess the impact of cluster-specific CpG sites on clustering performance, we compared results obtained with and without their inclusion. Using only CpG sites with mixed membership (as defined by the intersection in the Venn diagram in Fig. 2e) decreased clustering accuracy by 10% in the discovery cohort and 5% in the replication cohort (Suppl Fig. 5a). However, implementing the set of 900 unique CpG sites (cluster-specific) resulted in only a 5% performance drop in the discovery cohort and no change in the replication cohort (Suppl Fig. 5b). All in all, both mixed and cluster-specific methylation markers show informative value.

Taken together, these results confirm the strong potential of these 1,557 DNA methylation markers to distinguish individuals belonging to the different high-risk clusters.

Next, we asked whether the identified 1,021 genes mapping to these 1,557 markers are also epigenetically regulated in metabolic tissues. Analysis of published methylome data revealed that 862 out of 1,021 genes exhibited altered DNA methylation across various tissues in individuals with T2D. In particular 635, 631, and 512 genes were differentially methylated in the pancreatic islets [30], adipose tissue [31], and skeletal muscle [32] of individuals with T2D (Suppl Fig. 6). However, data on tissue-specific epigenetic alterations are still limited, especially for studying prediabetes heterogeneity. Hence, to better understand the potential role of these epigenetic predictors, we followed a parallel approach and performed gene ontology (GO) analysis with 255, 206, 205, and 249 genes corresponding to the cluster-specific 317, 300, 316, and 349 CpGs, respectively (Fig. 2f, Table S2). Several genes related to cluster LR were involved in cellular signaling and function, however, were not significantly enriched in any GO terms. In cluster 3 (HR-LowBeta), several genes were linked to negative regulation of TGF-β receptor signaling (GO:0030512), cellular response to calcium ions (GO:0019722), and axon guidance (GO:0007411). Cluster 5 (HR-InsRes) is the only one in which genes related to MAPK signaling (GO:0043410) and ECM organisation are enriched, both known to play a role in metabolic dysfunction-associated steatotic liver disease (MASLD). Osteoblast differentiation (GO:0001649) was also specifically enriched in cluster 5 (HR-InsRes). However, all the genes included in this GO term (*SFRP1*, *IGFBP5*, *LEF1*, *SOX8*, *RUNX2*) were previously linked to kidney development and/or diseases. The majority of the genes examined in cluster 6 (HR-InsSecr) were linked to Wnt and SMAD signaling, cell proliferation and cell-to-cell adhesion (Fig. 2f, Table S3). Notably, multiple genes were previously linked to kidney diseases [33, 34]. As CpG annotations rely only on physical proximity to genes, future functional experiments examining the impact of DNA methylation at these CpG sites on gene expression will be conducted in the near future. Finally, the identified methylation markers, mirroring the different future complications of the clusters, could partially explain the metabolic heterogeneity of the clusters.

### Screening EWAS data revealed shared epigenetic signatures with T2D-related traits

**Fig. 3:**
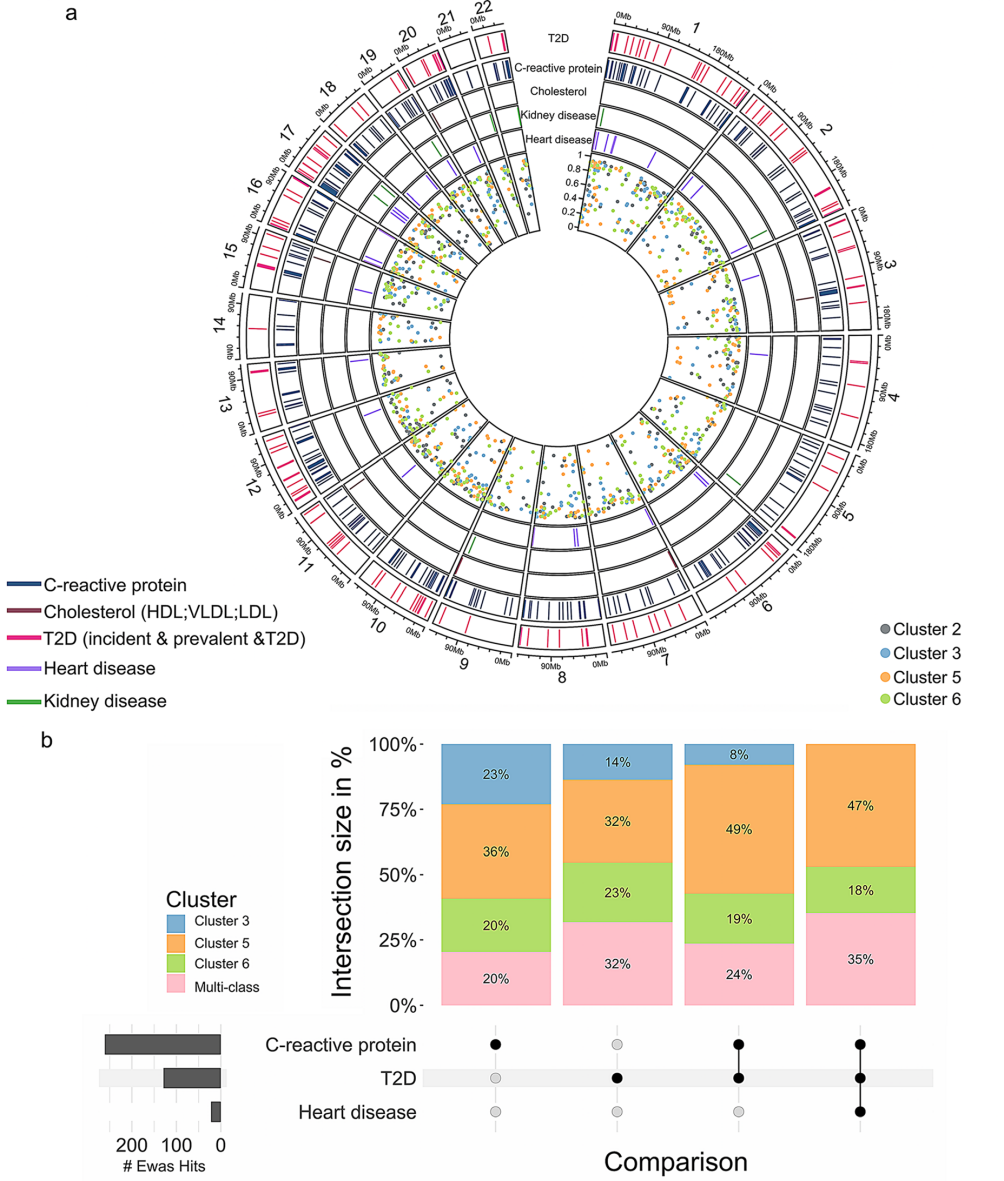
Cluster 5 shares most epigenetic signatures with previous EWAS studies. (**a**) The overlap between cluster-specific CpG sites and EWAS data is illustrated in the circos plot. The genomic positions of each CpG site are indicated with colored dots in the inner circle, while the five outer circles, marked with solid lines, represent the corresponding overlap with EWAS data. Each line refers to a cluster-specific CpG previously associated with heart and kidney diseases, cholesterol levels, CRP levels and T2D incidence. (**b**) The intersection plot summarizes the data presented in (**a**), additionally including CpG sites with mixed membership. Each bar represents the overlap between the 1,557 CpG sites and those previously linked to CRP, T2D incidence, CRP & T2D, CRP & T2D & heart disease, respectively. Bars are divided into cluster-specific and mixed-membership CpGs and are scaled to 100%. The bottom left bar plot (dark grey) shows the total number of CpG sites overlapping with EWAS findings; CpGs exclusively linked to one trait are marked by a black dot, and those associated with multiple traits are connected with black lines

Overlapping the list of the identified 1,557 predictors with blood cell DNA methylation data of the EWAS catalogue (details are in supplementary methods) resulted in 373 CpGs previously associated with traits linked to T2D and/or its complications. For instance, we identified 160 CpGs linked to T2D incidence, 11 to kidney diseases, and 32 to heart disease. Next, we examined specific traits related to obesity and T2D of which 331, nine, and eight were associated with C-reactive protein (CRP), BMI and, blood lipids, respectively (Table S4). Few CpGs appeared in several EWAS, for example, 100 CpGs were linked to both T2D incidence and CRP and 20 CpGs were associated with CRP, T2D incidence and cardiovascular disease. Similar results were found when examining cluster-specific CpGs (Fig. 3a). As shown in the intersection plot, the majority of CpGs detected in earlier EWAS overlapped with the cluster 5 (HR-InsRes) -specific CpG sites, especially those found in common for CRP, T2D and heart diseases (Fig. 3b). While we confirmed nearly 400 CpGs previously associated with T2D/related traits, this study also identified novel markers that can distinguish between high and low prediabetes clusters.

### Correlation of CpG predictors with anthropometric and metabolic traits

**Fig. 4:**
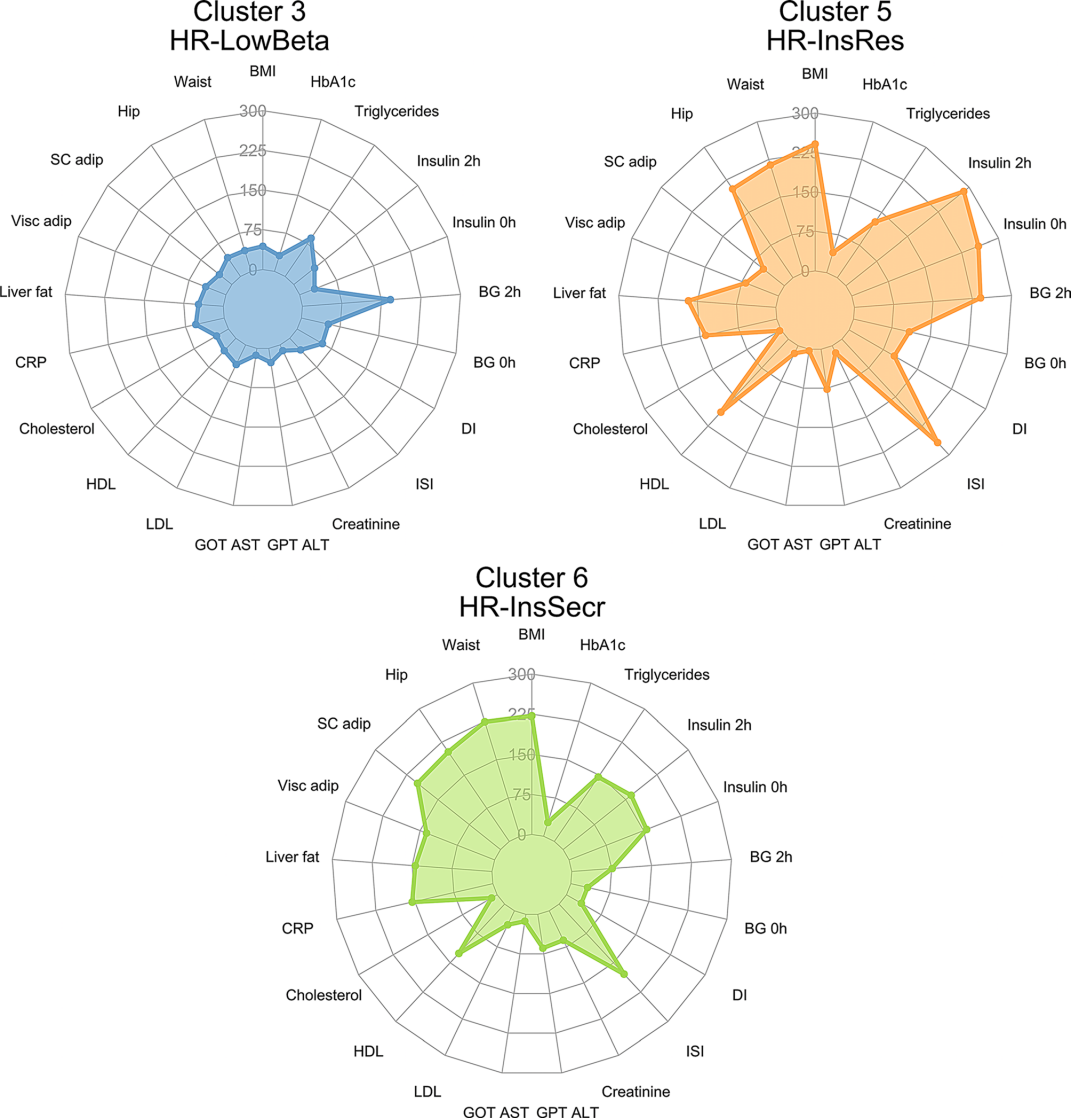
DNA methylation levels of cluster-specific CpG sites correlate to different anthropometric and metabolic parameters. Spearman correlations between DNA methylation levels at cluster-specific CpG sites and metabolic data are summarized in a spider plot. The value on each radial axis indicates the number of CpGs correlated with the corresponding phenotype. Abbreviations: ISI Matsuda (ISI), disposition index (DI), fasting blood glucose before OGTT (BG 0 h), blood glucose levels after 2 h of OGTT (BG 2 h) MR-tomography-based measurements of subcutaneous adipose tissue (SC adipo), MR-tomography-based measurements of visceral adipose tissue (Visc adipo), ^1^H-MR-spectroscopy-based measurements of hepatic lipid content (IHL) and liver enzymes: aspartate aminotransferase (GOT-AST), alanine transaminase (GPT-ALT)

In an attempt to understand the molecular differences between the high-risk clusters, we specifically examined the correlations between the cluster-specific CpGs with each anthropometric and metabolic measurement (detailed in Table 1). As presented in Fig. 4, cluster 3 (HR-LowBeta) had the highest number of CpGs correlating with blood glucose levels after OGTT, followed by triglycerides, LDL, and creatinine levels. In cluster 5 (HR-InsRes), the number of CpGs correlating with insulin and glycemic traits was more than 200 (e.g. 268 CpGs correlated with ISI Matsuda values, 241 CpGs with blood glucose during OGTT) followed by a considerable number of correlations with blood lipids (e.g. 351 CpGs for triglycerides). Of note, cluster 6 (HR- InsSecr) characterized by severe obesity and low T2D risk, exhibited only few CpGs correlating with blood glucose levels; however, it showed the highest number of CpGs correlating with MR-tomography-based measurements of subcutaneous adipose tissue and ISI Matsuda (Fig. 4). All in all, the identified methylation markers were specifically correlated with the most stringent metabolic characteristics of each cluster. These findings provide additional evidence that epigenetic markers found in blood cells have the potential to substitute classic clinical parameters used in clustering methods to identify individuals at risk for T2D.

## Discussion

Identifying individuals at high-risk of developing T2D and/or complications is an ultimate goal to achieve a better prognosis to start timely interventions. In an earlier study, we discovered six different prediabetes clusters, Tübingen Diabetes Risk Clusters, by analysing data of anthropometric measurements, MRI and biomarkers of insulin sensitivity, secretion and glycemia of about 900 people [11]. Here, we describe a machine learning workflow which allows to distinguish between individuals from three clusters with high-risk for T2D (clusters 3, 5, 6) only by the detection of 1,557 DNA methylation sites in blood cells.

Importantly, these results obtained in a discovery cohort (*n* = 187) were successfully replicated in an independent cohort composed of 146 individuals. Between 300 and 349 CpG sites have a unique methylation pattern in each cluster. Gene ontology analysis demonstrated that genes linked to the cluster-specific CpGs are involved in distinct biological processes, indicating that these epigenetic markers may contribute to the molecular differences between the T2D risk groups.

In cluster 3 (HR-LowBeta), which is characterized by a relatively low insulin secretion and high polygenic risk score for beta cell failure, biological processes of cellular response to calcium and TGF-β signaling appear to be affected, both previously linked to kidney and atherosclerotic cardiovascular disease (ASCVD) [35, 36], which are future complications in this cluster. Several candidates were already described to be involved in ASCVD such as *PRDM16* [36, 37], and *HTRA1* [35, 38]. PRDM16 was designated as key transcriptional factor required to maintain the myocardial cardiomyocyte identity but also to play an important role in myocardial metabolism. In mice with T2D, the cardiac-specific deletion of *Prdm16* led to aggravated cardiac dysfunction, exacerbated apoptosis, impaired mitochondria function, and increased cardiac lipid accumulation [36]. *HTRA1*, another cluster 3-specific candidate encoding a serine protease, facilitates collagen detachment from ER and contributes to myocardial fibrosis in dilated cardiomyopathy.

In cluster 5 (HR-InsRes), characterized by obesity, elevated liver fat content, and insulin resistance, we detected genes associated to MAPK signaling, which regulates hepatic lipid metabolism and glucose homeostasis through phosphorylation of downstream targets [39]. The osteoblast differentiation GO term was specifically enriched in cluster 5 and included genes implicated in kidney development and diseases, which are also potential future complications for this cluster, but different from the candidates associated with cluster 3 (HR-LowBeta) and 6 (HR-InsSecr). Not only individuals from cluster 5 (HR-InsRes), but also those belonging to cluster 6 (HR-InsSecr) had high-risk to develop severe nephropathy. Indeed, several cluster-specific methylation markers were located in genes linked to SMAD and Wnt signaling, both known to play an important role in kidney dysfunction [40–42].

Although GO analysis shows enrichment of these methylation markers in regulatory elements of genes involved in metabolic pathways associated with the clinical differences among the four clusters, these findings should be interpreted cautiously, as direct functional evidence linking CpG methylation to gene expression is lacking in the current study.

The correlation analysis suggested additional potential molecular differences between the clusters. Notably, several traits related to insulin secretion and resistance were correlated with DNA methylation levels at cluster 3-specific CpGs annotated to genes involved in islet function. For example, *Rab27a* mediates the tight docking of insulin granules onto the plasma membrane during glucose stimulation [43]. While both *CDK5*, *KCNQ1* were previously linked to beta cell apoptosis and loss, *CDK5* plays another pivotal role in the process of insulin secretion. Upon stimulation with high glucose, *CDK5* regulates the Ca^2+^ influx across the membrane [44] and contributes in the microtubule disassembly in beta cells [45].

A large number of genes were identified by GWAS to be associated with prediabetes and T2D, however, these genetic signals fail to detect and define the clinical or metabolic heterogeneity of the individuals. In contrast, we demonstrated that epigenetic signatures can distinguish between the prediabetes clusters without including clinical data. The epigenome is influenced by the environment (early life exposure, dietary intake, physical activity and others), which may be mirrored in blood cells. We hypothesize that the unhealthy lifestyle, captured by the DNA methylation in blood cells, is a major driver for the development of prediabetes and T2D, explaining why the epigenome is a better proxy than genetic factors. Accordingly, it is well accepted that early maternal exposures such as gestational diabetes, pre-gestational obesity, and famine but also physical activity are linked to altered DNA methylation patterns [46–48] and affect the metabolism of the offspring. In addition, physical activity, dietary interventions and weight loss are sufficient to remodel the epigenome of adults [49, 50].

In a similar context as the current study, it has been shown that specific epigenetic patterns in blood cells are linked to T2D clusters, including SIRD, SIDD, MARD, and MOD. Each cluster had distinct methylation risk scores that were also associated with increased or decreased risk of future complications, particularly cardiovascular disease and nephropathy [51]. Clinical parameters to assign the clusters are based on highly standardized workflows in specialized research units. These data are derived from oral glucose tolerance tests which, beside glucose measurements, necessitate the detection insulin concentrations and complex calculations for estimating insulin sensitivity and first-phase insulin secretion. Oral glucose tolerance tests in these standardized settings are not part of routine clinical work-ups, are cost- and time-intensive and often not covered by health insurances. On the contrary, only one blood draw is needed for assessing DNA methylation and considering profoundly reduced costs in the near future, these more easily accessible parameters provide a cost- and time-saving alternative to assess T2D and complication risk.

This study has limitations: (i) the discovery and replication cohorts are from German ancestry and thus conclusion maybe limited to individuals of Central European ancestry. (ii) While machine learning usually relies on high sample numbers, the cohort size of the present study was limited to 187 individuals (discovery cohort). To overcome the reduced statistical power and to enhance the stability of features (CpGs), a multi-fold cross-validated E-net was applied and results were then validated in an independent cohort from different center. (iii) Additional functional studies will be needed in the future to establish causality. Although the identified CpG markers are biologically plausible and enriched for relevant pathways, direct experimental validation of their functional consequences such as gene expression modulation in different cell types derived from metabolic tissues or animal models is warranted in future studies. (iv) Blood DNA methylation profiling provides a snapshot of stable epigenetic markers, however, connecting these changes to the pathophysiological status of a specific tissue is challenging. To support the assumption that DNA methylation in blood cells can mirror changes that occur in metabolically relevant tissues, a multi-tissue DNA methylation atlas could be a valuable tool for the current study. However, such data are not available so far. (v) Although DNA methylation in blood cells is known to be stable over time [52, 53], the present study might benefit from longitudinal methylation screening.

In conclusion, by applying a machine learning approach we identified epigenetic signatures in blood cells specifically for those prediabetes clusters which are at high-risk of developing diabetes and complications. These epigenetic signatures also suggest distinct molecular mechanisms underlying the disease’s heterogeneity, particularly across high-risk clusters.

Clinically, the identification of blood-borne biomarkers for the prediabetes offers the advantage of eliminating the need for time- and resource-intensive tests, such as the oral glucose tolerance test. This would enable the application of the risk stratification approach to broader populations.

## Supplementary Information

Below is the link to the electronic supplementary material.

Supplementary Material 1

Supplementary Material 2

Supplementary Material 3

Supplementary Material 4

Supplementary Material 5

Supplementary Material 6

## Data Availability

The majority of the data are included in the supplementary Tables, and all the raw methylation data will be available in the GEO repository GSE315764. Clinical data are available from the corresponding author on reasonable request.

## Abbreviations

CpG: Cytosine-phosphate-guanine
T2D: Type 2 diabetes
HbA1c: Glycated haemoglobin
LR: Low risk
HR-LowBeta: High risk low beta cell function
HR-InsRes: High risk high insulin resistance
HR-InsSecr: High risk high insulin secretion
PLIS: Prediabetes Lifestyle Intervention Study
TUEF: Tuebingen Family Study
TULIP: Tuebinger Lebensstil-Interventionsprogramm
IFIS: Intermittent fasting to improve insulin secretion
IQR: Interquartile range
SVD: Singular value decomposition
βvalue: Beta value (methylation)
E-net: Elastic net regularization
PAM: Partitioning around medoid
TSS: Transcription start site
EWAS: Epigenome wide association studies
BMI: Body-mass index
VAT: Visceral adipose tissue
IHL: Intrahepatic lipid
DMP: Differentially methylated positions
ISI Matsuda: Matsuda insulin sensitivity index
OGTT: Oral glucose tolerance test
HDL: High-density lipoprotein
LDL: Low-density lipoprotein
UMAP: Uniform manifold approximation and projection
GO term: Gene ontology term
MASLD: Metabolic dysfunction-associated steatotic liver disease
CRP: C-reactive protein
ASCVD: Atherosclerotic cardiovascular disease
GWAS: Genome-wide association studies
SIRD: Severe insulin-resistant diabetes
SIDD: Severe insulin-deficient diabetes
MARD: Mild age-related diabetes
MOD: Mild obesity-related diabetes
