# Supplementary material for: Stratifying high-risk prediabetes clusters using blood-based epigenetic markers
Source: Biomark Res. 2026 Jan 17;14:19. doi: 10.1186/s40364-025-00887-8 (PMC12829285; doi:10.1186/s40364-025-00887-8)
Supplement: Supplementary file 1 — Supplementary Material 1 [file 40364_2025_887_MOESM1_ESM.pdf]

a

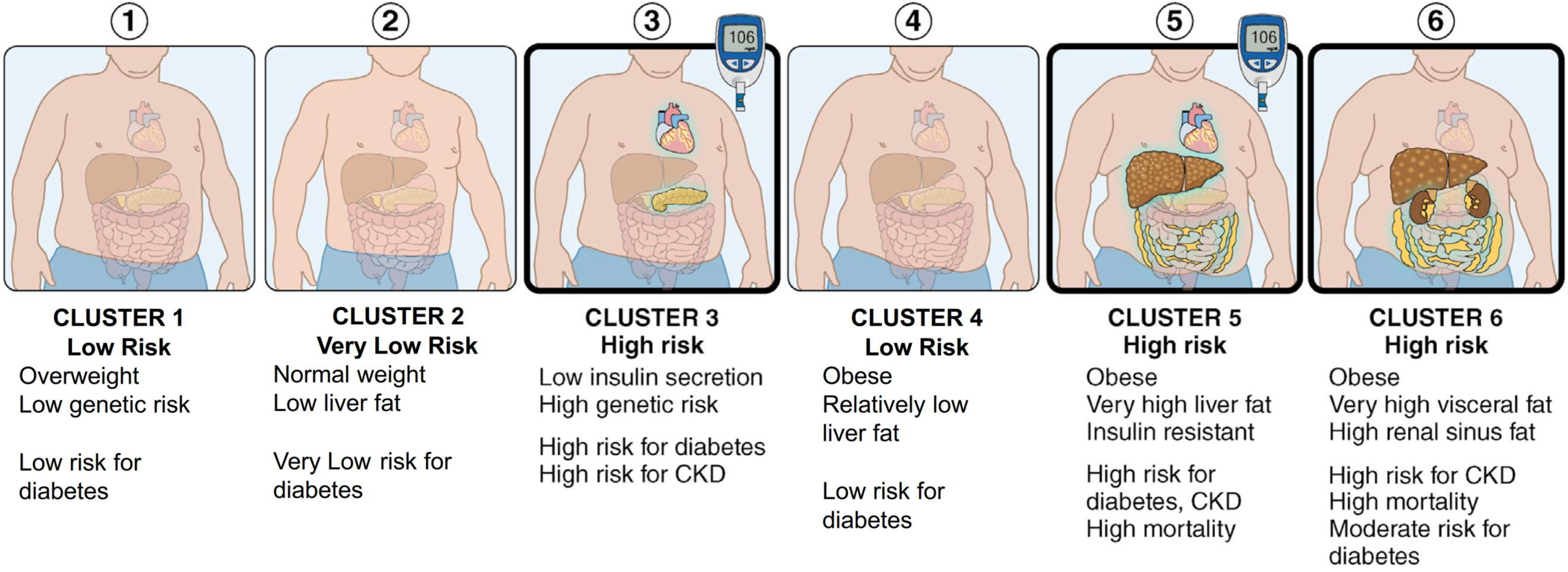

Supplementary Figure 1

b

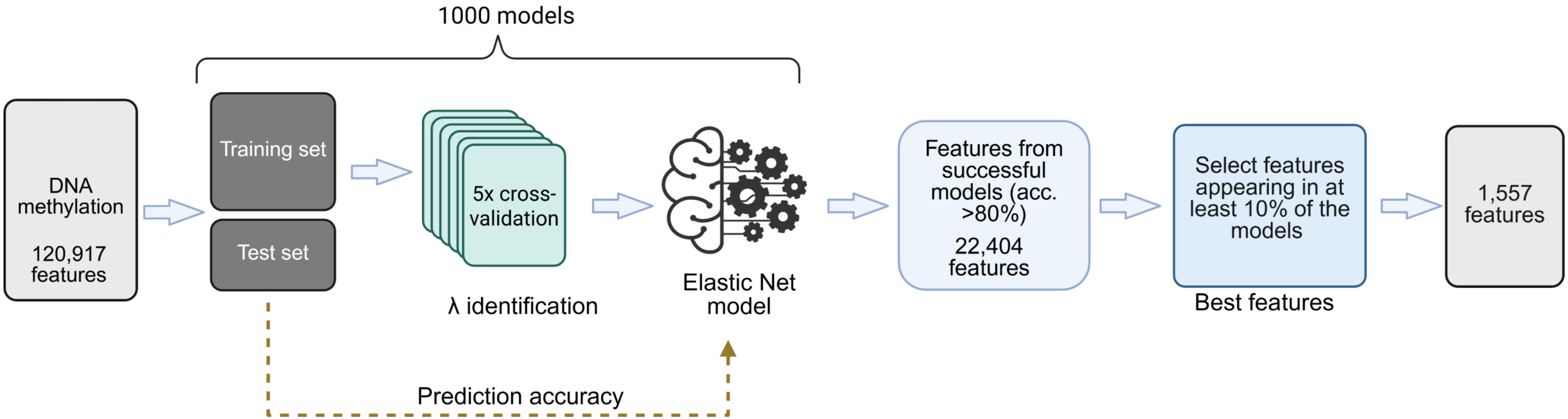

Supplementary Figure 1

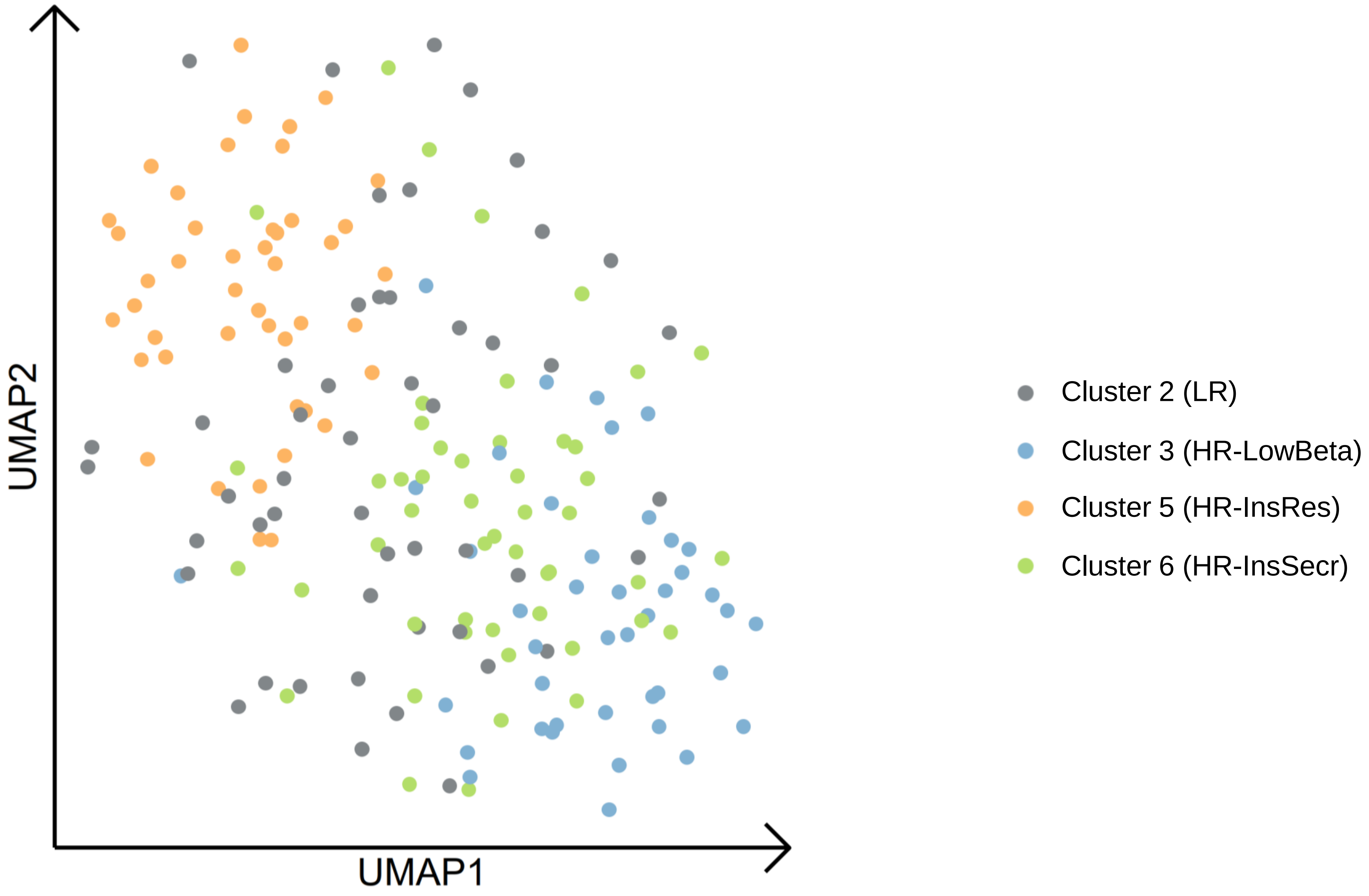

Supplementary Figure 2

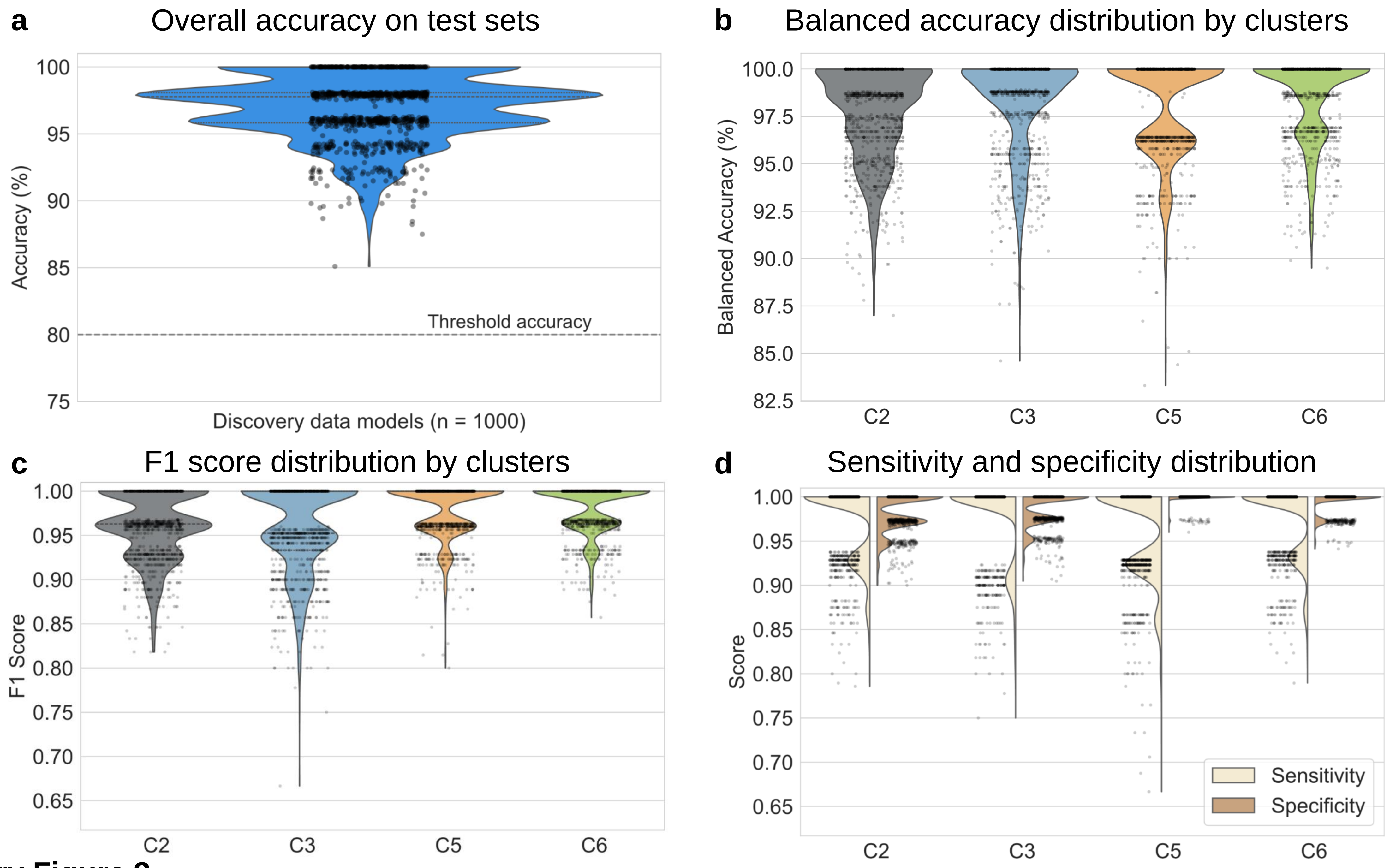

Supplementary Figure 3

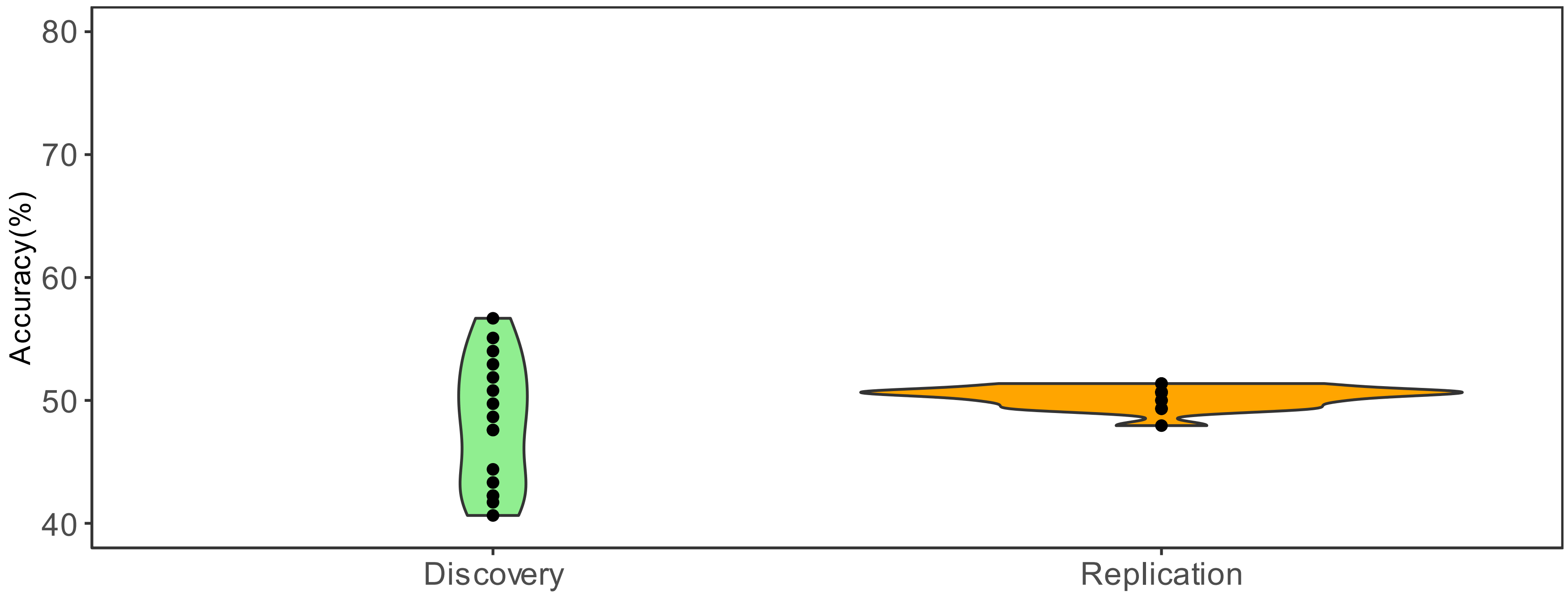

Supplementary Figure 4

a

Mixed membership

Discovery Cohort (68.4%)

Prediction \ Actual	C2	C3	C5	C6
C2	24	3	10	2
C3	8	33		8
C5	16	4	35	4
C6	2	2		36

Replication Cohort (89%)

Prediction \ Actual	C3	C5	C6
C3	44	6	1
C5		43	1
C6	5	2	44

b

Cluster-specific

Discovery Cohort (73.2%)

Prediction \ Actual	C2	C3	C5	C6
C2	32	1	18	2
C3	7	36		
C5	9	4	26	5
C6	2	1	1	43

Replication Cohort (93.1%)

Prediction \ Actual	C3	C5	C6
C3	45	2	
C5	1	46	1
C6	3	3	45

Supplementary Figure 5

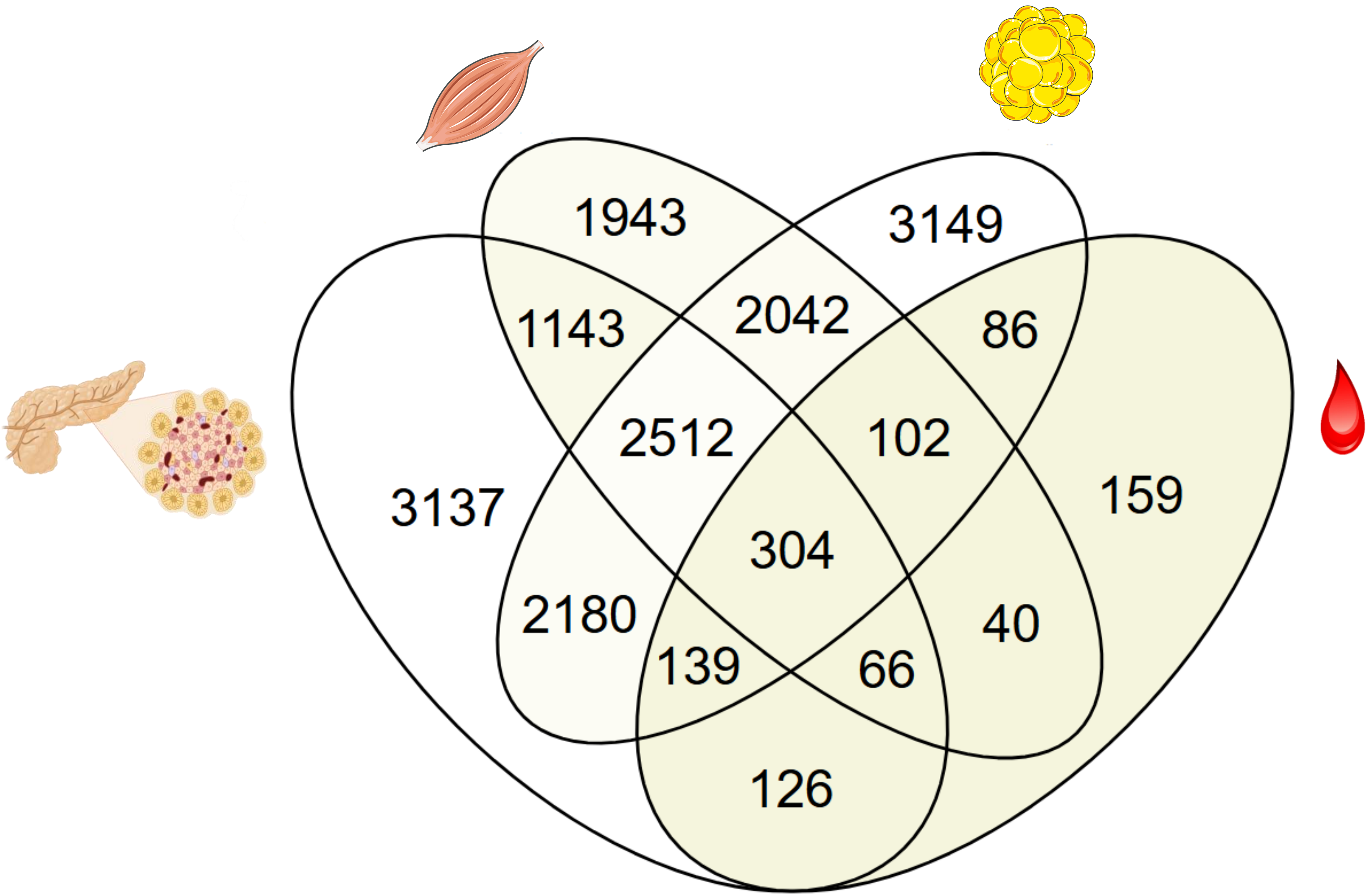

Supplementary Figure 6
