# Supplementary material for: Stratifying high-risk prediabetes clusters using blood-based epigenetic markers
Source: Biomark Res. 2026 Jan 17;14:19. doi: 10.1186/s40364-025-00887-8 (PMC12829285; doi:10.1186/s40364-025-00887-8)
Supplement: Supplementary file 2 — Supplementary Material 2 [file 40364_2025_887_MOESM2_ESM.docx]

**Supplementary Information**

**Supplementary Figures**

**Figure S1. Detailed bioinformatics workflow**

(a) This figure is adapted from our previous publication [1] and illustrates the main metabolic and clinical differences between the six clusters. (b)Feature selection process using an E-net model. Starting with 120,917 DNA methylation features (also DMP), the dataset is split into training and testing sets. Next, 1000 E-net models undergo 5-fold cross-validation on the training set to identify the optimal λ (lambda), and mixing penalty α (alpha) was identified using a grid search using caret – an R package which was used in all models. In total, 22,404 features (CpGs) were generated by these 1000 models. Features appearing in at least 10% of the all models achieving >80% accuracy are selected, resulting in a refined set of 1,557 features for further analysis.

**Figure S2. All detected differentially methylated CpGs cannot distinguish the clusters sufficiently.** (A) The UMAP plot shows the heterogenous distribution of the 120,917 DMPs between the clusters. The color codes refer to cluster identity.

**Figure S3. Performance distributions of 1,000 e-net models on test sets from the discovery cohort**. (a) A violin plot showing the distribution of the overall classification accuracy of all 1,000 models in the held-out test samples of the discovery cohort. Each point represents the performance of a single model in the test samples. The dashed horizontal line indicates the 80% accuracy threshold required for a model to be considered successful for feature retention. (b) violin plots showing the distribution of balanced accuracy, (c) the distribution of F1 score, stratified by each of the four clusters (LR: cluster 2, HR-LowBeta: cluster 3, HR-InsRes: cluster 5, HR-InsSecr: cluster 6). (d) Split violin plots showing the distribution of sensitivity (beige) and specificity (brown) for each cluster.

**Figure S4. Distribution of clustering accuracy using randomly selected CpGs shows low accuracy.** The violin plots depict accuracies obtained from PAM clustering of fifteen randomly selected 1,500 CpGs in discovery (left) and replication (right) cohorts.

**Figure S5. CpGs with mixed cluster memberships have informative value in clustering high risk clusters.** Results of the partition around medoids clustering approach comparing predicted (methylation driven) and actual (phenotype driven) cluster identity based on mixed membership (a) and cluster-specific CpG sites (b). The agreement between methylation and phenotype-driven clustering is shown in the blue rectangles. Light blue rectangles refer to the disagreement between the two clustering.

**Figure S6. Genes detected by E-net are epigenetically regulated in different metabolic tissues of individuals with T2D.** Venn diagram showing the overlap of multi-tissue methylome results collected from previous publications [2–4] and the 1,021 of E-net. Number refer to the number of genes of each comparison.

**Supplementary Methods**

***Study population description***

Glucose regulation was assessed by a 5-point oral glucose tolerance test [5], body fat distribution, i.e. subcutaneous adipose tissue and visceral adipose tissue by whole-body MRI [5] and hepatic lipid content (IHL) by ^1^H-MR-spectroscopy [6]. Prediabetes was defined according to criteria of the American Diabetes Association (ADA) with elevated fasting glucose 100-125 mg/dL (from 5.6 to 6.9 mmol/L) and/or a 2h glucose of 140-199 mg/dL (from 7.8 to 11.0 mmol/L). Laboratory assessment was implemented as previously described [7]. From the OGTT, indexes of insulin sensitivity (ISI Matsuda) and beta cell function (Disposition Index: ISI x Insulinogenic Index) were calculated as previously described [8,9].

***References for supplementary methods:***

1. Sandforth L, Kullmann S, Sandforth A, Fritsche A, Jumpertz-von Schwartzenberg R, Stefan N, et al. Prediabetes remission to reduce the global burden of type 2 diabetes. Trends Endocrinol Metab [Internet]. 2025 [cited 2025 May 15]; Available from: https://pubmed.ncbi.nlm.nih.gov/39955249/

2. Rönn T, Ofori JK, Perfilyev A, Hamilton A, Pircs K, Eichelmann F, et al. Genes with epigenetic alterations in human pancreatic islets impact mitochondrial function, insulin secretion, and type 2 diabetes. Nat Commun [Internet]. 2023 [cited 2025 Jul 11];14:8040. Available from: https://pmc.ncbi.nlm.nih.gov/articles/PMC10716521/

3. Nilsson E, Vavakova M, Perfilyev A, Säll J, Jansson PA, Poulsen P, et al. Differential DNA Methylation and Expression of miRNAs in Adipose Tissue From Twin Pairs Discordant for Type 2 Diabetes. Diabetes [Internet]. 2021 [cited 2025 Jul 11];70:2402–18. Available from: https://dx.doi.org/10.2337/db20-0324

4. Ling C, Vavakova M, Ahmad Mir B, Säll J, Perfilyev A, Martin M, et al. Multiomics profiling of DNA methylation, microRNA, and mRNA in skeletal muscle from monozygotic twin pairs discordant for type 2 diabetes identifies dysregulated genes controlling metabolism. BMC Med [Internet]. 2024 [cited 2025 Jul 11];22:572. Available from: https://pmc.ncbi.nlm.nih.gov/articles/PMC11613913/

5. Fritsche A, Wagner R, Heni M, Kantartzis K, Machann J, Schick F, et al. Different Effects of Lifestyle Intervention in High- and Low-Risk Prediabetes: Results of the Randomized Controlled Prediabetes Lifestyle Intervention Study (PLIS). Diabetes [Internet]. 2021 [cited 2025 May 27];70:2785–95. Available from: https://dx.doi.org/10.2337/db21-0526

6. Machann J, Thamer C, Schnoedt B, Stefan N, Haring HU, Claussen CD, et al. Hepatic lipid accumulation in healthy subjects: A comparative study using spectral fat-selective MRI and volume-localized 1H-MR spectroscopy. Magn Reson Med [Internet]. 2006 [cited 2025 May 27];55:913–7. Available from: /doi/pdf/10.1002/mrm.20825

7. Wagner R, Heni M, Tabák AG, Machann J, Schick F, Randrianarisoa E, et al. Pathophysiology-based subphenotyping of individuals at elevated risk for type 2 diabetes. Nat Med [Internet]. 2021 [cited 2025 May 15];27:49–57. Available from: https://www.nature.com/articles/s41591-020-1116-9

8. Matsuda M, DeFronzo RA. Insulin sensitivity indices obtained from oral glucose tolerance testing: Comparison with the euglycemic insulin clamp. Diabetes Care [Internet]. 1999 [cited 2025 May 15];22:1462–70. Available from: https://pubmed.ncbi.nlm.nih.gov/10480510/

9. Seltzer HS, Allen EW, Herron AL, Brennan MT. Insulin secretion in response to glycemic stimulus: relation of delayed initial release to carbohydrate intolerance in mild diabetes mellitus. J Clin Invest [Internet]. 1967 [cited 2025 May 15];46:323–35. Available from: https://pubmed.ncbi.nlm.nih.gov/6023769/
